# Pyramid urethral mobilisation hypospadias repair for distal variant: experience with 70 cases

**DOI:** 10.3389/fped.2025.1652994

**Published:** 2026-02-12

**Authors:** Mircea Andriescu, Olivia Stanciu, Iulia Stoicescu, Laura Balanescu, Radu Balanescu

**Affiliations:** 1 Department of Pediatric Surgery, “Grigore Alexandrescu” Emergency Hospital for Children, Bucharest, Romania; 2 Department of Paediatric Surgery and Orthopaedics, “Carol Davila” University of Medicine and Pharmacy, Bucharest, Romania

**Keywords:** autograft, distal hypospadias, pyramid dissection, redo hypospadias, urethral advancement

## Abstract

**Introduction:**

Choosing the appropriate surgical procedure for distal hypospadias is challenging due to the numerous techniques currently used. Urethral mobilization is an adjunctive surgical technique that aims to improve the position of the urethral meatus. We present our experience with a technique of urethral advancement in 70 cases of distal hypospadias.

**Materials and methods:**

We retrospectively reviewed the charts of patients with hypospadias for whom pyramid urethral advancement was performed between 2022 and 2025 in our center.

**Technique description:**

Dissection and mobilization of the native urethra along with the corpus spongiosum from the penile shaft—the dissection is performed in a pyramidal manner, addressing all planes of the native urethra along with the attached corpus spongiosum after transection of the lateral attachments of the “V-shaped” spongiosum from the base of the glans; mobilization of the urethra and corpus spongiosum “en bloc” along the corpus cavernosum thus exposing the inter-cavernosum groove. Next, a longitudinal, median incision is made in the avascular plane between the two corpus cavernosum in which the urethra-spongiosum complex is inserted and advanced distally. The results were satisfactory with good functional and cosmetic outcome and 4 patients who presented meatal stenosis.

**Discussion:**

The technique we propose combines elements from multiple procedures. The main rationale for this approach is to correct the defect using the child's native urethra and avoid a suture urethroplasty with its complications. Pyramid urethral mobilization can also successfully be applied in hypospadias redo cases if inclusion criteria are met. The main amendment in the procedure we performed is the longitudinal, median incision made in the avascular plane between the two corpus cavernosum. Conclusion. Urethral advancement by pyramid repair is a valid option for distal hypospadias and redo cases but precise case selection is mandatory when considering this procedure.

## 1 Introduction

With an estimated incidence ranging from 1 in 200 to 1 in 300 live male births, hypospadias constitutes one of the most prevalent urogenital malformations encountered in pediatric surgical practice (1). The primary objective of hypospadias repair is to restore both functional and anatomical normalcy, encompassing the creation of a vertically slit-like meatus at the tip of a straightened penis, while ensuring unobstructed urinary flow and minimizing long-term psychosocial sequelae. Over the past several decades, a plethora of surgical techniques have been proposed, refined, and debated—ranging from single-stage procedures such as the tabularized incised plate (TIP) urethroplasty, to multi-stage reconstructions for more proximal or complex variants. Hypospadias repair for distal variant is considered most challenging because of the numerous techniques published until present. The concept of urethral mobilization is not new; in fact, it dates back more than a century. Beck et al. (2) first introduced urethral mobilization in 1898, describing a technique for balanic hypospadias in which the distal urethra was dissected and advanced to improve meatal position. Subsequent authors expanded on this principle. McGowan et al. (3) reported mobilization of the anterior urethra as a safe and effective method for distal advancement. Barcat also incorporated urethral mobilization into his hypospadias repairs, influencing many later modifications.

In 1994, Koff et al. (4) described what he termed a “modified Barcat technique”, emphasizing extensive mobilization of the urethral plate and urethra, thereby popularizing the method but not originating it. Since then, multiple authors have continued to refine or revisit urethral mobilization and advancement for distal hypospadias. Türken et al. (5) reported excellent outcomes using an eccentric circumferential meatal-based flap combined with limited urethral mobilization. Haberlik et al. (6) described favorable long-term results using a modification of Beck's original operation. More recent experiences reaffirm the relevance of urethral mobilization and advancement as a versatile technique for selected distal cases (7, 8). These historical contributions provide the foundation on which our modified pyramid urethral advancement technique is based.

In 1981 Professor Stephen Koff et al. (9) published a paper considering the principle of total urethral mobilization as an adjunctive surgical technique for improving the position of the urethral meatus in hypospadias repair. Although the results were promising, the editors at the time invoked the difficulty of the urethral dissection when considering the small advancement distance obtained and the possibility of secondary curvature and meatal stenosis due to glans tunnelisation.

Eight years later Duckett and Keating (10) introduced the pyramid procedure for a particular form of hypospadias—mega meatus intact prepuce (MIP). In this technique the urethral plate and the distal native urethra are dissected in all planes, but the plate is tubularized.

In the same year the GAP procedure was presented (Glans Approximation Procedure) by Mark Zaontz (11) for hypospadias with deep glandular groove. The next publication that specifically approached distal variant hypospadias repair by urethral mobilization and advancement appeared twenty years later—GUD (Glandular Urethral Disassembly) proposed by Antonio Macedo (12).

The pyramid advancement technique we perform combines elements from all these procedures. The main rationale for this approach is to correct the defect using the child's native urethra and avoid a suture urethroplasty with its potential complications. The aim of this retrospective study is to introduce a variant of urethral advancement technique we perform in our center and present our results this procedure over the last three years.

## 2 Materials and methods

### 2.1 Study design and population

This retrospective study included 70 boys with hypospadias who underwent surgical repair at our institution between January 2022 and May 2025.

### 2.2 Eligibility criteria

Inclusion Criteria:

Patients were eligible if they met all of the following criteria:

– hypospadias type: distal hypospadias. In this study, distal ectopic meatus refers to a meatal location at the subcoronal or distal penile shaft level, without involvement of the midshaft or proximal segments.
– penile curvature: mild curvature identified intraoperatively after complete penile degloving using an artificial erection test.
– chordee severity was measured intraoperatively using a goniometer during artificial erection and classified as: mild: <20°; mild to moderate: 20–30°; moderate: 30–45°; moderate to severe: >45°. Only patients with residual curvature <30° after complete penile degloving were included. Patients with persistent curvature ≥30° were excluded and managed with alternative hypospadias repair techniques requiring formal chordee correction.
– redo hypospadias cases with distal ectopic meatus and no residual curvature were also included.
– all included patients underwent repair using the pyramid advancement procedure.

Exclusion Criteria:

Patients were excluded if they had:

– moderate or severe penile curvature
– proximal hypospadias
– glandular hypospadias

### 2.3 Follow-up

Patients were followed for a minimum of 3 months and a maximum of 24 months, with a median follow-up duration of 12 months. Fifty-eight patients (82.8%) were followed for more than 6 months. Thirty-nine patients (55.7%) had follow-up exceeding 12 months.

### 2.4 Outcome measures

Surgical success was assessed using functional, cosmetic, and patient/parent-reported outcomes.

Functional Outcomes:

– straight penis with no residual chordee
– normal urinary stream without spraying or straining
– patent neourethra without fistula or stricture

Cosmetic Outcomes:

– urethral meatus located at or near the tip of the glans
– symmetrical glans and penile shaft
– natural penile appearance without significant scarring

Patient/Parent Satisfaction: subjective satisfaction with penile appearance and function was assessed during follow-up, with particular attention to minimizing psychological distress during adolescence.

### 2.5 Functional assessment

Objective uroflowmetry was not routinely available due to the young age of many patients. Functional outcomes were therefore evaluated clinically based on: straight urinary stream without deviation or spraying; absence of straining or dysuria; absence of recurrent urinary tract infections; patent urethral meatus confirmed by calibration during follow-up; these assessment criteria were applied uniformly to all patients.

### 2.6 Ethical approval

The study was approved by the institutional ethics committee. Written informed consent was obtained from the parents or legal guardians of all patients prior to surgery.

### 2.7 Statistical analysis

Statistical analysis was performed using IBM SPSS Statistics for Mac. Descriptive statistics were used to summarize patient demographics, operative details, and outcomes. Continuous variables are presented as medians with ranges, while categorical variables are reported as absolute numbers and percentages. Given the retrospective, single-arm design of the study, no comparative or inferential statistical analyses were performed.

### 2.8 Surgical technique

After treatment of preputial adhesions, a 5–0 PGA suture is placed in the dorsum of the glans to facilitate handling during subsequent repair. Objective evaluation of ventral penile curvature was performed (using either a spontaneous erection at the induction of anesthesia or induced erection with saline solution) by goniometry.

**Figure 1:**
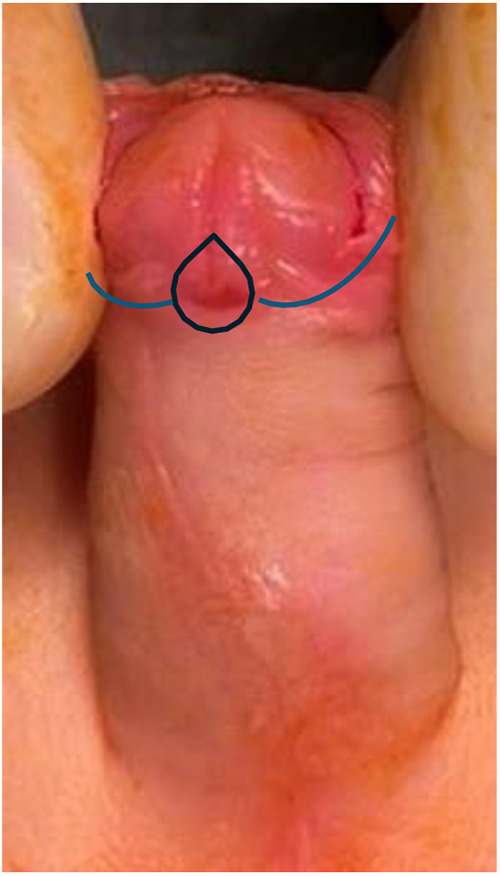
Drop-shaped incision around the ectopic meatus. The incision outlines the base of the pyramid dissection and defines the distal limit of mobilization.

**Figure 2:**
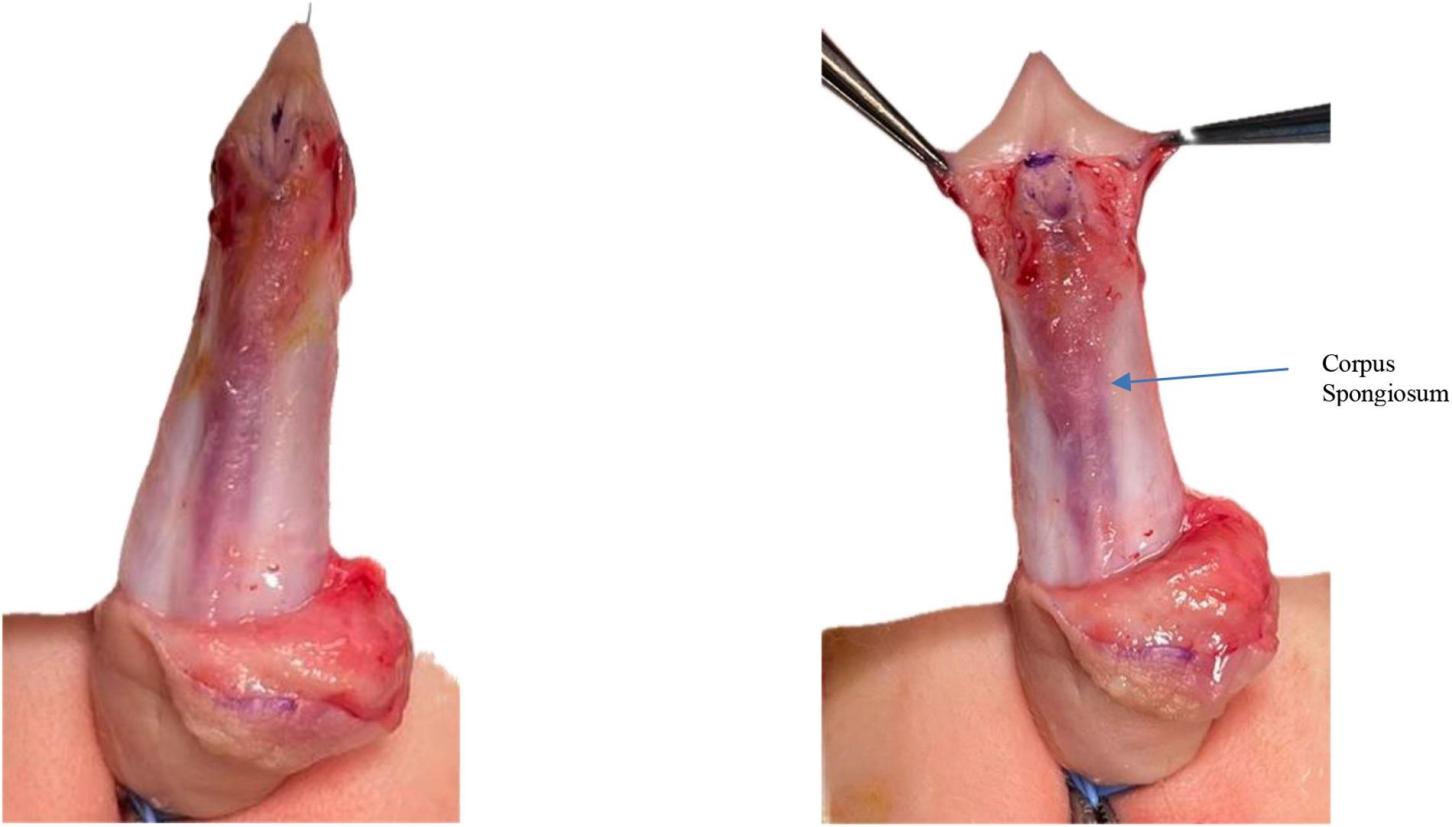
Penile degloving performed through the avascular plane between Buck's and dartos fascia to correct ventral curvature.

A “drop-shaped” incision is made around the ectopic urethral meatus and is continued on the lateral and posterior aspect of the prepuce, circumferentially, at approximatively 5 mm below the corona (Figure 1). Penile degloving to the penoscrotal junction is done to correct the ventral curvature with dissection in the avascular plane between Buck's fascia and dartos fascia; care must be taken when separating the ventral skin from the often hypoplastic underlying urethra to avoid injury to the urethra (Figure 2).

**Figure 3:**
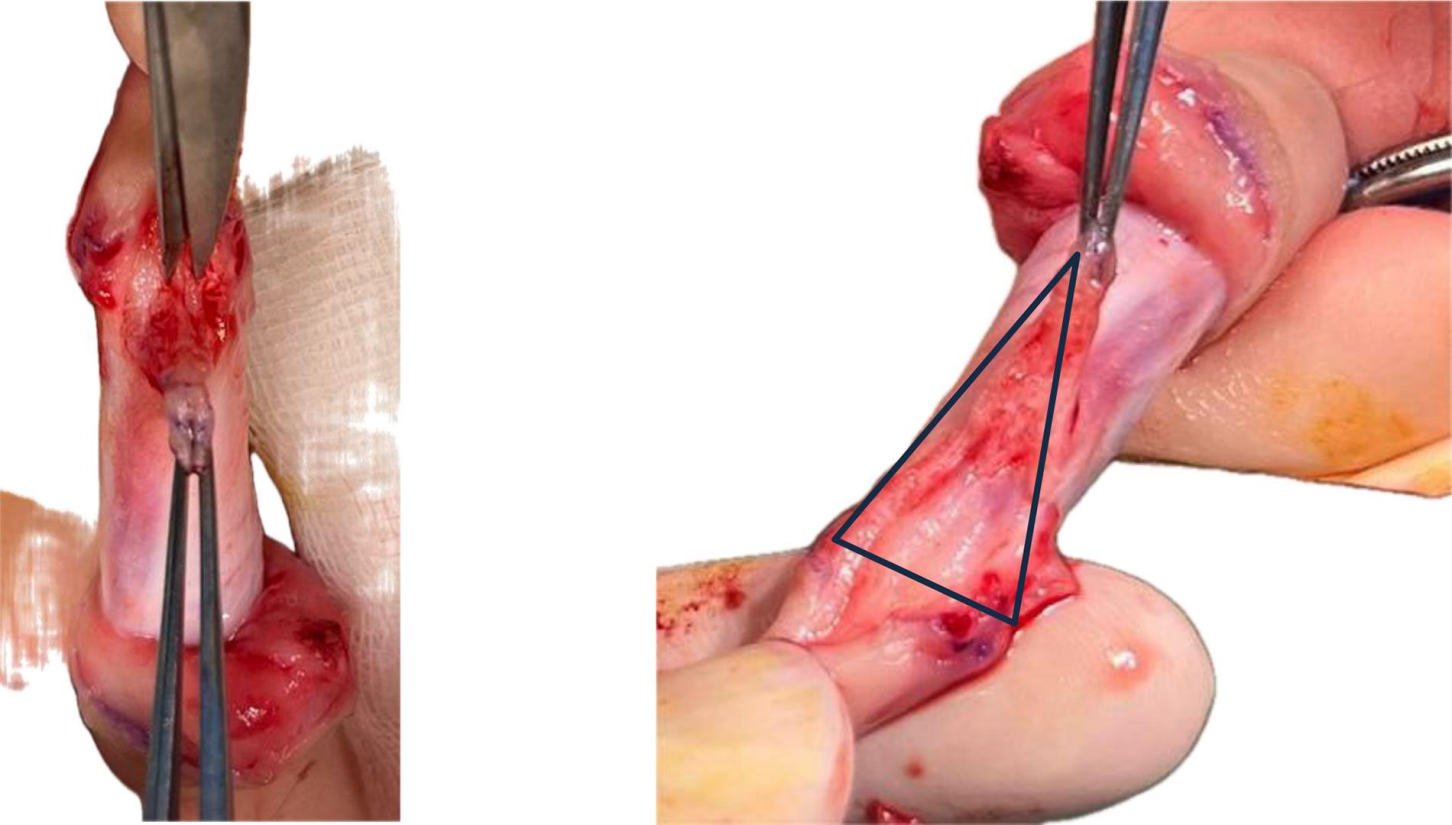
Mobilization of the urethra–spongiosum complex from the corporal bodies.

Dissection and mobilization of the native urethra with the corpus spongiosum from the penile shaft is performed in a pyramidal manner, addressing all planes of the native urethra along with the attached corpus spongiosum after transection of the lateral attachments of the “V-shaped” spongiosum from the base of the glans; the next step is the mobilization of the urethra and corpus spongiosum “en bloc” along the corpus cavernosum thus individualizing the inter-cavernosum groove (Figure 3). At this point bleeding from small vessels penetrating the inter-cavernous septum can occur but should not be mistaken for corporeal bleeding. The proximal extent of urethral mobilization was determined intraoperatively by following the urethra–spongiosum complex until both lateral bulbar spongiosa arteries were clearly identified, ensuring preservation of the vascular pedicle. Mobilization was performed proximally only to the point at which the urethra could be advanced distally to the glans without tension. The adequacy of lengthening was assessed by aligning the mobilized urethra along a fully stretched penis; only when the urethra reached the glans tip without traction was advancement performed. If this criterion could not be met despite further proximal mobilization, the patient was not considered suitable for the pyramid advancement technique.

**Figure 4:**
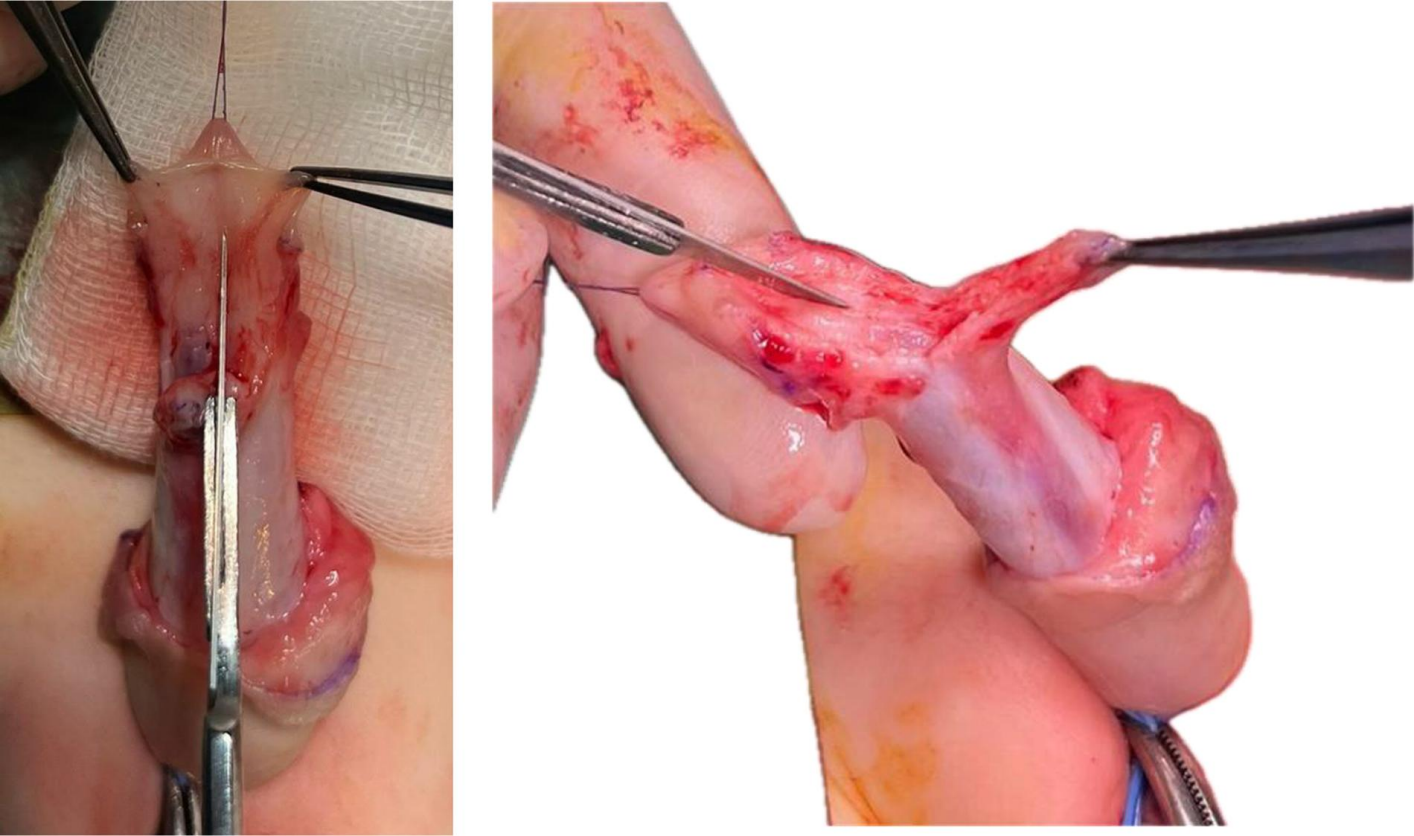
Longitudinal incision in the inter-cavernosum avascular plane creating a sulcus for tension-free advancement.

**Figure 5:**
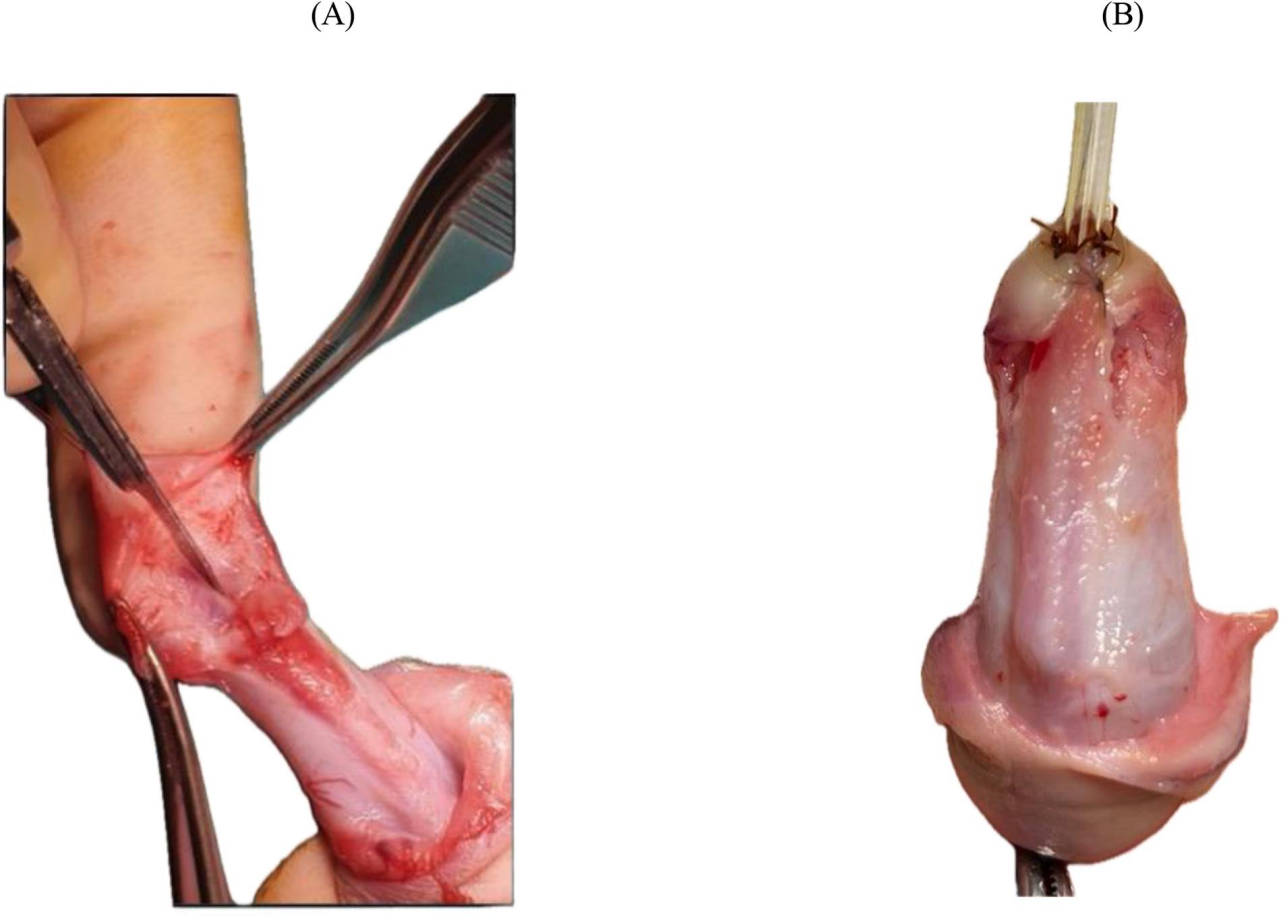
Advancement and fixation of the urethra–spongiosum complex. **(A)** Mobilized urethra–spongiosum complex before advancement. **(B)** Advancement and fixation of the complex to the glans tip using 6-0 absorbable sutures.

Inferior tilting of the entire complex reveals the triangular shape. The separated urethra-spongiosum complex is now measured against the taut phallus to determine whether a tensionless anastomosis to the tip of the glans is feasible. Next, a longitudinal, median incision is made in the avascular plane between the two corpus cavernosum from the inferior limit of the disassembled native urethra to the tip of the glans—a sulcus is obtained in which the urethra-spongiosum complex is slightly elongated, placed and advanced distally; the depth of the sulcus is appreciated intraoperatively considering the size of the urethra-spongiosum complex (Figure 4). The urethra is anastomosed to the tip of the glans with 6/0 absorbable sutures in 6 points (Figure 5). A catheter is passed through into the bladder and left indwelling.

**Figure 6:**
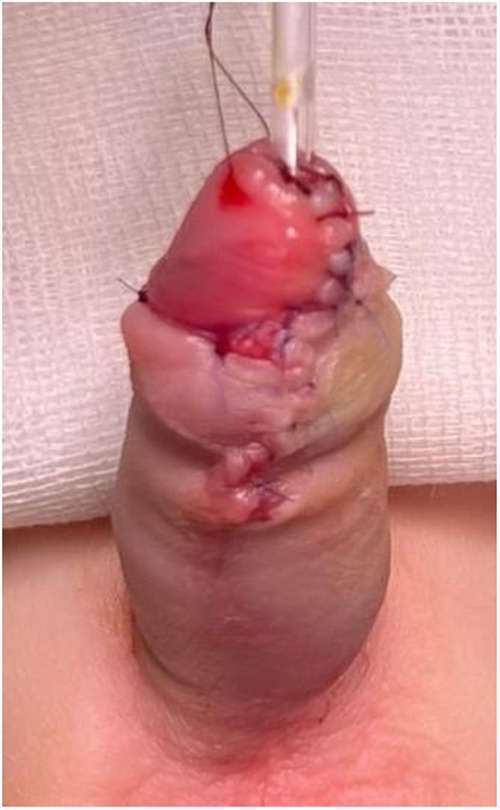
Glansplasty and final cosmetic appearance with the meatus positioned at the glans tip.

The lateral wings of the glans are brought together over the urethra with three subcutaneous 6/0 absorbable sutures concluding the glansplasty (Figure 6). The ventral penile shaft is resurfaced in any fashion depending on the quality and quantity of the prepuce.

An appropriately sized catheter is left indwelling for approximately 7 days; the catheter is secured with a 5/0 resorbable suture to the glans. Slightly compressive dressing is used with sterile dressing covered in iodine, antibiotic ointment and self-adhesive gauze.

## 3 Results

### 3.1 Patient characteristics

A total of 70 hypospadias repairs were performed using the described technique at “Grigore Alexandrescu” Emergency Hospital for Children between 2022 and 2025. Sixty-five patients presented with coronal hypospadias associated with a mild degree of chordee, while five patients were redo cases with distal ectopic meatus and no curvature. All redo cases labeled as having a distal ectopic meatus had a meatal position between the subcoronal margin and the distal third of the penile shaft. None of the patients had penile torsion. The median age at the time of surgery was 3.6 years.

### 3.2 Operative and perioperative data

The median duration of the surgical procedure was one hour. A urethral catheter was maintained for seven days, and the first dressing change was performed after four days. The median length of hospital stay was seven days. No intraoperative complications were reported in the operative records. One patient experienced catheter obstruction in the immediate postoperative period.

### 3.3 Follow-up

The median duration of follow-up was 12 months.

### 3.4 Functional outcomes

At the one-month follow-up, all patients demonstrated good functional outcomes, with all boys voiding with a straight urinary stream and showing no residual chordee. Four patients developed meatal stricture during the medium-term follow-up period (3–6 months), for which meatoplasty was performed. At the one-year follow-up, all patients had satisfactory functional outcomes.

### 3.5 Cosmetic outcomes

At the one-year follow-up evaluation, all patients demonstrated good cosmetic outcomes.

## 4 Discussion

### 4.1 Historical context and rationale for technique modification

The origins of urethral mobilization can be traced back to Beck's 1898 description, where distal urethral advancement was introduced as a method for correcting balanic hypospadias (6). Over the decades, several authors revisited and refined this concept. McGowan (1964) further demonstrated that the anterior urethra could be safely mobilized to achieve a more orthotopic meatal position (3). Barcat later incorporated similar principles into his urethral repairs, establishing a widely adopted foundation for distal advancement techniques (4). Although Koff's 1994 publication, titled “modified Barcat technique”, helped popularize urethral mobilization in modern hypospadias surgery, it is clear from the historical record that he was not the first to describe this approach. Additional refinements have been contributed by Haberlik et al., Türken et al., and more recently Hassan, and Edan, all of whom reported good functional and cosmetic outcomes with variations of urethral mobilization and advancement in distal hypospadias (5–8). These cumulative contributions highlight that urethral mobilization is a long-standing surgical principle with multiple documented evolutions, not a novel concept. Our technique builds on this historical foundation while introducing a distinct modification—creation of a longitudinal inter-cavernosum sulcus to permit tension-free advancement of the native urethra–spongiosum complex.

As Stephen Koff already mentioned in his publication, rather than being a specific type of hypospadias repair, mobilization of the anterior urethra is an adjunctive surgical technique for improving the position of the meatus and is adaptable to a variety of hypospadias conditions (9). The pyramid exposure provides a safe and simple dissection of the urethral plate and allows subsequent caliber with excellent functional and cosmetic results (10). Mark Zaonts described a glandular approximation procedure for glandular and coronal hypospadias with a wide, deep glandular groove (11). The corpus spongiosum and urethra are supplied by bulbourethral arteries, one on either side, which are the branches of the internal pudendal arteries. Hence, the urethra with corpus spongiosum can be safely mobilized to the level of the penoscrotal junction without fear of de-vascularization (13). The postoperative healing process involves an inflammation response with vascular endothelial growth factors so one must consider an anastomosis of the corpus spongiosum to the glans in addition to the urethral mobilization. The technique we propose combines elements from all these procedures. The main rationale for this approach is to correct the defect using the child's native urethra and avoid a suture urethroplasty with its potential complications (14).

### 4.2 Technical innovation and distinctive features

The innovation of our approach lies in the longitudinal, median incision created in the avascular inter-cavernosum plane, which enables distal advancement of the urethra–spongiosum complex without tension or compromise of vascular supply. This maneuver differs from previously described methods such as Koff's urethral mobilization, Duckett's pyramid repair for the mega-meatus intact prepuce variant, and Macedo's glandular urethral disassembly (GUD). By combining the principles of safe urethral mobilization with a controlled inter-cavernosum release, our modification facilitates tension-free advancement while maintaining the native urethral integrity and minimizing suture-related complications.

Thus, the procedure refines and extends the applicability of existing advancement concepts rather than introducing an entirely new principle.

### 4.3 Patient selection and surgical considerations

What the history of hypospadias repair has taught us is that no procedure fits all (15). The surgical technique should be decided intraoperatively and customized accordingly after degloving the penis and observing the true extent of the defect. Furthermore, the selected method may not be sufficiently appropriate, potentially leading to the patient requiring a repeat procedure. Therefore, it is our strong opinion that careful patient selection is the most important step when considering urethral advancement (15). Patients with moderate or severe chordee are not candidates for this technique because even with good dissection of the native urethra you cannot achieve a tension-free suture to the tip of the glans. The length of the advancement after urethral mobilization is smaller than the length of the degloved penis. In cases of mild to moderate curvature the procedure of pyramid advancement is appliable and also, we observed that the disassembly of the urethra-spongiosum complex aids to the correction of the chordee.

Another important factor is the clinical aspect the glans which should not be considered as an exclusion criteria for this technique. If the lateral wings of the glans are extensively prepared, as per Snodgrass TIP procedure (16), the subsequent glanduloplasty should not compress the advanced urethra.

Patients that needed testosterone pre-op for undersized penis were not candidates for this procedure. Redo cases represent a special category of hypospadias patients. This procedure can successfully be applied in these situations if the inclusion criteria are met, but most importantly, the scar tissue must be excised entirely before starting the urethral dissection.

### 4.4 Clinical outcomes in comparison with established techniques

When compared with published results of established distal hypospadias repairs, our outcomes appear comparable (Table 1). In large TIP urethroplasty series, reported fistula rates range from 5%–10% and meatal stenosis from 3%–8%. In our cohort, the incidence of meatal stenosis was 5.7% (4 of 70 cases), with no fistula or glans dehiscence observed. Although a direct control group was not included, this suggests that pyramid urethral mobilization yields functional and cosmetic results within the expected range of current standards while offering the advantage of avoiding neourethral suture lines. A prospective comparative analysis would be valuable to confirm these observations.

**Table 1:** Comparison with other techniques from the literature.

Technique	Key concept	Reported complications	Comments/advantages
Koff (9)	Total urethral mobilization	Limited distal reach; risk of tension	First to use urethral advancement
Duckett and Keating (10)	Pyramid repair for MIP	Specific indication only	Safe dissection in all planes
Macedo (20)	GUD disassembly	Technically demanding	Allows precise glans remodeling
Present technique	Pyramid mobilization with inter-cavernosum incision	Meatal stenosis 5.7%	Simpler, tension-free advancement preserving native urethra

Urethral advancement by pyramid repair is an option for distal hypospadias but careful case selection is of foremost importance when considering this procedure. In order for this procedure to become standardized large prospective cohort studies are needed.

### 4.5 Complications and pathophysiological considerations

Four cases presented meatal stenosis in the medium term follow-up period. A meatoplasty was performed in all cases and no other complications developed after this procedure. Partial retraction of the advanced native urethra is a documented complication of mobilization-advancement techniques (17). We believe that this is the mechanism for meatal stenosis in the four cases who developed medium-term complications.

Advancement of the native urethra in hypospadias patients is not a new technique, variations of the procedure have been published through the years (18, 19). The main amendment in the procedure we performed is the longitudinal, median incision made in the avascular plane between the two corpus cavernosum from the inferior limit of the disassembled native urethra to the tip of the glans obtaining a sulcus in which the urethra-spongiosum complex is placed and advanced distally. When correctly performed, this longitudinal incision does not injure the corpora and contributes to both the tension free suture of the urethra-spongiosum complex to the glans and a smooth healing process with no retraction of the advanced native urethra. In conclusion, the native urethra—spongiosum advanced complex is comparable to an autograft.

The median follow-up period of 12 months represents a limitation, as certain complications such as urethral stricture or meatal stenosis may develop several years postoperatively. However, most early and intermediate complications of hypospadias repair typically manifest within the first postoperative year. Ongoing follow-up of this patient cohort is underway to document long-term functional and cosmetic outcomes beyond the current observation window.

### 4.6 Study limitations

This study has several limitations. First, its retrospective, single-center design limits the level of evidence and introduces potential selection bias. Second, the absence of a control group prevents definitive comparison with other established techniques such as TIP or GUD urethroplasty. Third, the follow-up duration is relatively short to assess late complications. Nevertheless, all procedures were performed by the same surgical team using a uniform technique, ensuring internal consistency of results. Future multicenter prospective studies with extended follow-up and objective functional evaluation are warranted to validate the long-term benefits of pyramid urethral mobilization.

### 4.7 Future directions and clinical implications

The findings of this study suggest that pyramid urethral mobilization represents a safe and effective alternative for selected cases of distal hypospadias and redo repairs. By emphasizing the use of the child's native urethra and avoiding urethral tabularization, this modification simplifies the reconstructive process while minimizing suture-related complications. The addition of a longitudinal incision in the inter-cavernosum avascular plane provides a reproducible method for achieving tension-free advancement and anastomosis, potentially reducing the risk of postoperative curvature or ischemic compromise.

Looking forward, several avenues for development are evident. A multicenter prospective trial with standardized inclusion criteria and objective outcome measures would be valuable to validate the reproducibility of this approach. Incorporating validated scoring systems such as the Hypospadias Objective Scoring Evaluation (HOSE) or the Pediatric Penile Perception Score (PPPS) could enhance the comparability of results across studies. Long-term follow-up beyond puberty is essential to assess urinary and sexual function, penile growth, and patient satisfaction, as certain complications may only become apparent over time.

From a clinical standpoint, pyramid urethral mobilization may serve as an intermediate option between traditional advancement and neourethral reconstruction, especially in distal or redo cases where the urethral plate remains adequate. With further refinement and broader validation, this approach could contribute to a more individualized, anatomy-preserving strategy in modern hypospadias surgery.

## 5 Conclusion

Urethral advancement by pyramid mobilization represents a valuable option for selected cases of distal hypospadias and redo surgery. The addition of a longitudinal inter-cavernosum incision allows safe and tension-free advancement of the native urethra–spongiosum complex with satisfactory early outcomes. While the results are promising, larger prospective comparative studies and extended follow-up are required to establish this modification as a standardized technique within the spectrum of distal hypospadias repairs.

## Data Availability

The datasets presented in this article are not readily available because our datasets contain private information about the patients and we only have their consent to use privately for statistical purposes. Requests to access the datasets should be directed to oliviastefan89@gmail.com.
